# CMR derived left ventricular septal convexity in carriers of the hypertrophic cardiomyopathy-causing *MYBPC3*-Q1061X mutation

**DOI:** 10.1038/s41598-019-42376-7

**Published:** 2019-04-11

**Authors:** Mika Tarkiainen, Petri Sipola, Mikko Jalanko, Tiina Heliö, Pertti Jääskeläinen, Kati Kivelä, Mika Laine, Kirsi Lauerma, Johanna Kuusisto

**Affiliations:** 1 Department of Radiology, Kuopio University Hospital, Kuopio, Finland; 2 Heart and Lung Center, Department of Cardiology, Helsinki University Hospital and University of Helsinki, Helsinki, Finland; 3 Heart Centre, Kuopio University Hospital, Kuopio, Finland; 4 Department of Radiology, Helsinki University Central Hospital, Helsinki, Finland; 5 Centre for Medicine and Clinical Research, University of Eastern Finland and Kuopio University Hospital, Kuopio, Finland

## Abstract

This manuscript has not been published before and is not currently being considered for publication elsewhere. Increased septal convexity of left ventricle has been described in subjects with hypertrophic cardiomyopathy (HCM) -causing mutations without left ventricular hypertrophy (LVH). Our objective was to study septal convexity by cardiac magnetic resonance (CMR) in subjects with the Finnish founder mutation Q1016X in the myosin-binding protein C gene (*MYBPC3)*. Septal convexity was measured in end-diastolic 4-chamber CMR image in 67 study subjects (47 subjects with the *MYBPC3*-Q1061X mutation and 20 healthy relatives without the mutation). Septal convexity was significantly increased in subjects with the *MYBPC3*-Q1061X mutation and LVH (n = 32) compared to controls (11.4 ± 4.3 vs 2.7 ± 3.2 mm, *P* < 0.001). In mutation carriers without LVH, there was a trend for increased septal convexity compared to controls (4.9 ± 2.5 vs 2.7 ± 3.2 mm, *P* = 0.074). When indexed for BSA, septal convexity in mutation carriers without LVH was 2.8 ± 1.4 mm/m^2^ and 1.5 ± 1.6 mm/m^2^ in controls (*P* = 0.036). In all mutation carriers, septal convexity correlated significantly with body surface area, age, maximal LV wall thickness, LV mass, and late gadolinium enhancement. Subjects with the *MYBPC3*–Q10961X mutation have increased septal convexity irrespective of the presence of LVH. Septal convexity appears to reflect septal remodeling, and could be useful in recognizing LVH negative mutation carriers.

## Introduction

Hypertrophic cardiomyopathy (HCM) is the most common inherited cardiovascular disease and one of the most common causes of sudden cardiac death in the young^1^. HCM is caused by autosomal dominant mutations in genes encoding mainly sarcomeric proteins. More than 1400 mutations causing HCM have been identified in over 13 genes^2^. In familial HCM, there is a 50% chance that the first-degree relative will inherit the gene mutation leading to the disease. HCM is a heterogenous disease with variable clinical phenotype even in patients carrying the same causal mutation^3^. Clinical presentation may vary from asymptomatic to arrhythmia, heart failure and sudden death. Clinical manifestations of HCM usually develop during adolescence but myosin-binding protein C gene (*MYBPC3*) mutation carriers often have no LVH or develop LVH later in life^4–6^.

More than 40% of the mutations causing HCM are found in the *MYBPC3* gene^7^. The founder mutation Q1061X in *MYBPC3* (*MYBPC3*-Q1061X) is the most common single mutation causing HCM in Finland and is found in about 11% of Finnish HCM cases^5,8,9^. To our knowledge, this mutation is extremely rare outside Finland, with one reported mutation in non-Finnish populations according to the Genome Aggregation Database (gnomAD). This autosomal dominant mutation has a relatively low penetrance and often, but not always, mild phenotype^6,8^. Of the subjects with the Finnish founder mutation *MYBPC3*-Q1016X, 20 to 30% do not have LVH and are considered phenotype negative mutation carriers^6,8,10^.

Hypertrophic cardiomyopathy is characterized by asymmetric LV hypertrophy, most often located in the LV anterior septum or free wall. Of HCM patients with reverse septal curvature on echocardiography, nearly 80% have a positive genetic test for myofilament HCM, contrasting those with sigmoidal septal morphology, who have positive genetic test in fewer than 10% of cases^11^. Recently, CMR derived septal convexity into the LV has been suggested to be an additional, previously undescribed feature of subclinical HCM^12^. Compared to controls, abnormal convexity was significantly increased in phenotype negative carriers of HCM-causing mutations in multiple sarcomere genes. To our knowledge, there are no other studies regarding this topic. Therefore, the purpose of our study was to further investigate septal convexity by CMR in subjects carrying the single *MYBPC3*-Q1016X mutation and their healthy relatives.

## Methods

### Study population

All available subjects with the *MYBPC3*-Q1061X mutation and their healthy relatives from Kuopio and Helsinki University Hospital areas were prospectively recruited. Altogether, 74 adult subjects representing 25 families with the Finnish founder mutation *MYBPC3*-Q1061X were screened. The Genome Center of the University of Eastern Finland performed all the genetic studies as previously described^8^. All index patients of the present study were screened for 59 cardiomyopathy associated genes, and no other pathogenic or likely pathogenic mutations in addition to *MYBPC3-Q1061X* were found in any of the patients^9^. Seven subjects had implanted pacemaker precluding CMR imaging and were excluded from the study. The final study population consisted of 67 individuals (47 with the *MYBPC3*-Q1061X mutation with or without LVH, and 20 healthy relatives without the *MYBPC3*-Q1061X mutation).

The G+/LVH+ group consisted of 32 subjects with the *MYBPC3*-Q1061X mutation and no other cause for significant hypertrophy (LV maximal wall thickness [LVMWT] ≥ 13 mm in CMR), and 15 subjects with the same mutation but no hypertrophy (LVMWT < 13 mm in CMR) consisted the G+/LVH− group.

The study conforms to the principles outlined in the Declaration of Helsinki and the ethics committees at the Kuopio and Helsinki University Hospitals approved the study protocol. All subjects gave informed written consent.

### Echocardiography

All subjects underwent echocardiographic studies which were carried out by experienced cardiologists (J.K., P.J., M.J.) with GE Vivid 7 ultrasound equipment (GE Vingmed, Norway). Conventional measurements were performed according to AHA/ASE guidelines. A resting gradient >30 mmHg through the left ventricular outflow tract (LVOT) was considered significant^13^.

### CMR

CMR images were obtained with the same study protocol in both participating hospitals (Kuopio and Helsinki University Hospitals) as described previously^14^. Delayed-enhancement images were obtained 10 minutes after injection of gadoterate meglumine (Dotarem®) 0.2 mmol/kg contrast agent using segmented trueFISP IR sequence in short axis orientation and in two long axis orientations (4- and 2-chamber views). The imaging parameters were 700 msec temporal resolution, 1.08 msec echo time, flip angle 50°, acquisition matrix 192 × 144 and 340 × 340 field of view. Slice thickness was 8 mm and intersection gap 20%. Inversion time was optimized to null the signal intensity of normal myocardium.

### Image Analyses

**Figure 1:**
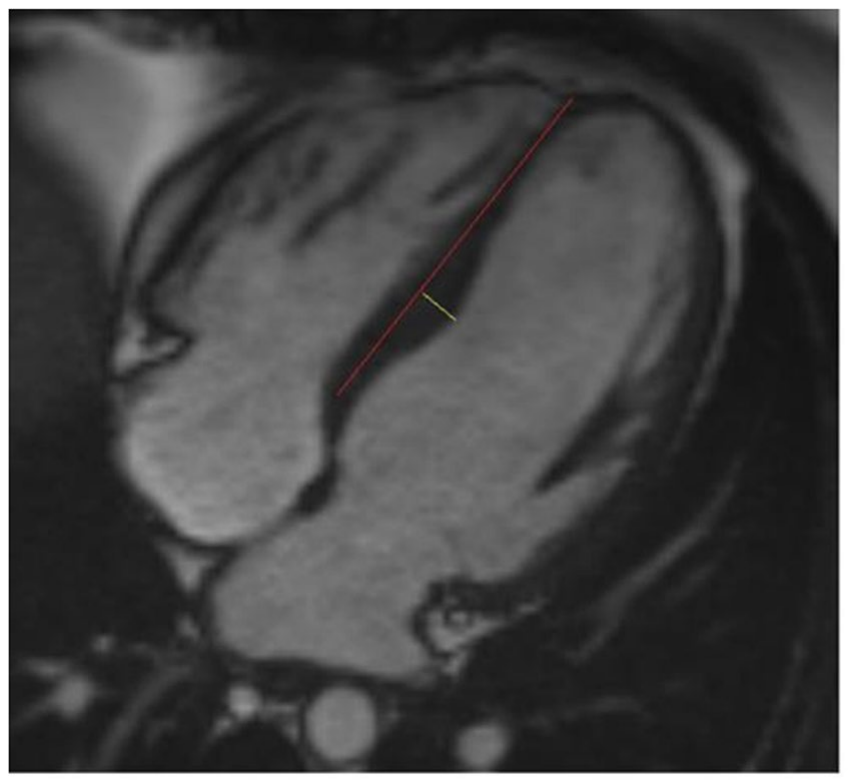
Measurement of septal convexity in end-diastolic 4-chamber CMR image. The red line is a reference line between the mid-wall at the level of tricuspid valve and the insertion point of the right ventricle into the left ventricle at the apex. The yellow line is the measured septal convexity. This particular subject has the measured septal convexity of 9 mm and left ventricular maximal wall thickness of 12 mm.

All of the anatomical measurements on CMR images were performed by using Sectra IDS7 workstation. Image analysis was performed by experienced analyzers with no data of the study subjects’ clinical or genetic findings. Septal convexity into the LV was measured in end-diastolic 4-chamber images as the maximal distance between LV septal endocardial border and a line connecting septal mid-wall points at the level of tricuspid valve insertion and at the level of apical right ventricular insertion on the LV as described previously^12^ [Fig. 1]. M.T. (with 5 years experience in CMR) performed all septal convexity measurements. All the other anatomical measurements of the CMR images were performed as described previously^14^. Areas of late gadolinium enhancement (LGE) were traced by QMass® late-enhancement analysis software (QMass® 7.2, Medis Medical Imaging Systems, Netherlands) in short axis images and quantified segmentally as absolute and relative mass in a standard 16-segment model. A threshold of +6 SD was used for detection of LGE^15^.

Measurement repeatability was evaluated by measuring septal convexity twice in 20 randomly selected subjects. M.T. tested intraobserver variability by re-measuring septal convexity more than 6 months after the initial measurement. Interobserver variability was calculated from the same 20 measurements by another radiologist (K.K.).

### Statistical Analyses

Data is presented as mean ± standard deviation. Many of the variables had skewed distribution and were compared with Kruskal-Wallis one-way analysis of variance and independent samples with the Mann-Whitney U test. Receiver operating characteristic (ROC) curve was constructed to test the ability of septal convexity to differentiate between mutation carriers without LVH and control subjects. Paired samples T-test and Pearson correlation coefficient was used in repeatability testing. All statistical analyses were performed with IBM SPSS Statistics V22.0. and were considered significant with a *P* < 0.05.

## Results

### Characteristics of the study groups

Table 1 shows baseline, echocardiographic and CMR characteristics of the three study groups. G+/LVH− subjects were younger and had lower BSA compared to other groups. There were more males were in the G+/LVH− group compared to other groups. G+/LVH− subjects were all in NYHA 1 class^14^. They did not have cardiac symptoms or clinical findings (data not shown).

**Table 1:** Baseline, echocardiographic and CMR characteristics.

	MYBPC control	MYBPC G+/LVH−	MYBPC G+/LVH+	*P*
Patients, n	20	15	32	
Age, y	46 ± 17	33 ± 16*	50 ± 11	**0.007**
Men, n	5 (25%)	3 (20%)	20 (63%)*	<**0.001**
BSA, m²	1.87 ± 0.28	1.75 ± 0.18*	1.94 ± 0.20	**0.004**
Height, cm	169 ± 10	167 ± 6	174 ± 9	**0.04**
Weight, kg	76 ± 24	68 ± 13	81 ± 14	**0.005**
BMI	27 ± 8	24 ± 4	27 ± 4	0.129
**NYHA, n**				
I	—	—	28 (88%)	
II	—	—	4 (13%)	
LVOT gradient, mmHg	7.1 ± 2.9	5.7 ± 1.8	8.5 ± 11.4	0.42
LVMWT, mm	9.6 ± 1.7	9.5 ± 1.6	22.1 ± 5.7*	<**0.001**
LVMI, g/m2	45 ± 9	49 ± 13	68 ± 21*	<**0.001**
LVEDVI, ml/m2	78 ± 13	78 ± 15	74 ± 14	0.55
LVESVI, ml/m2	32 ± 10	30 ± 7	28 ± 10	0.13
LVEF, %	60 ± 8	62 ± 5	63 ± 9	0.12
LGE,n	—	1	23*	<**0.001**
SC, mm	2.7 ± 3.2	4.9 ± 2.5	11.4 ± 4.3*	<**0.001**

MYBPC-control = healthy controls without *MYBPC3* mutation, MYBPC G+/LVH− = *MYBPC3* mutation carriers without LVH, MYBPC G+/LVH+ = *MYBPC3* mutation carriers with LVH in CMR, BSA = body surface area, BMI = body mass index, NYHA = New York Heart Association functional class, LVOT gradient = left ventricular outflow tract gradient, LVMWT = left ventricular maximal wall thickness, LVMI = left ventricular mass index, LVEDVI = left ventricular end-diastolic volume index, LVESVI = left ventricular end-systolic volume index, LVEF = left ventricular ejection fraction, LGE = late gadolinium enhancement, SC = septal convexity into the left ventricle. *Significance *P* < 0.05 between MYBPC groups and controls.

No septal interventions (myectomy or ablation) had been performed in any of the study subjects. There was one subject with significant LVOT obstruction (gradient >30 mmHg on echocardiography) in the G+/LVH+ group. However, the mean LVOT gradients did not differ significantly between the study groups.

CMR derived LVMWT and left ventricular mass index (LVMI) differed between study groups, as expected. Ventricular volume indices (LVEDVI and LVESVI) and LVEF did not differ between study groups. G+/LVH+ subjects had mostly moderate hypertrophy (LVMWT 22.1 ± 5.7 mm) located mainly in septum (72%) or anterior wall (25%). True apical hypertrophy was not found. No significant difference was found in LVMWT between G+/LVH− subjects and controls (9.5 ± 1.6 vs 9.6 ± 1.7 mm, *P* = 0.882). LVMI was significantly increased in G+/LVH+ group compared to other groups (68 ± 21 g/m^2^, *P* < 0.001), but not in G+/LVH− subjects compared to controls (49 ± 13 vs 45 ± 9 g/m^2^, *P* = 0.438). In the G+/LVH+ group, 23 subjects had LGE, as only one in the G+/LVH− group had LGE. In the subjects with LGE, the mean amount of LGE was 18 ± 12% of the LV myocardium (range 1–45%).

### Septal convexity into the LV

**Figure 2:**
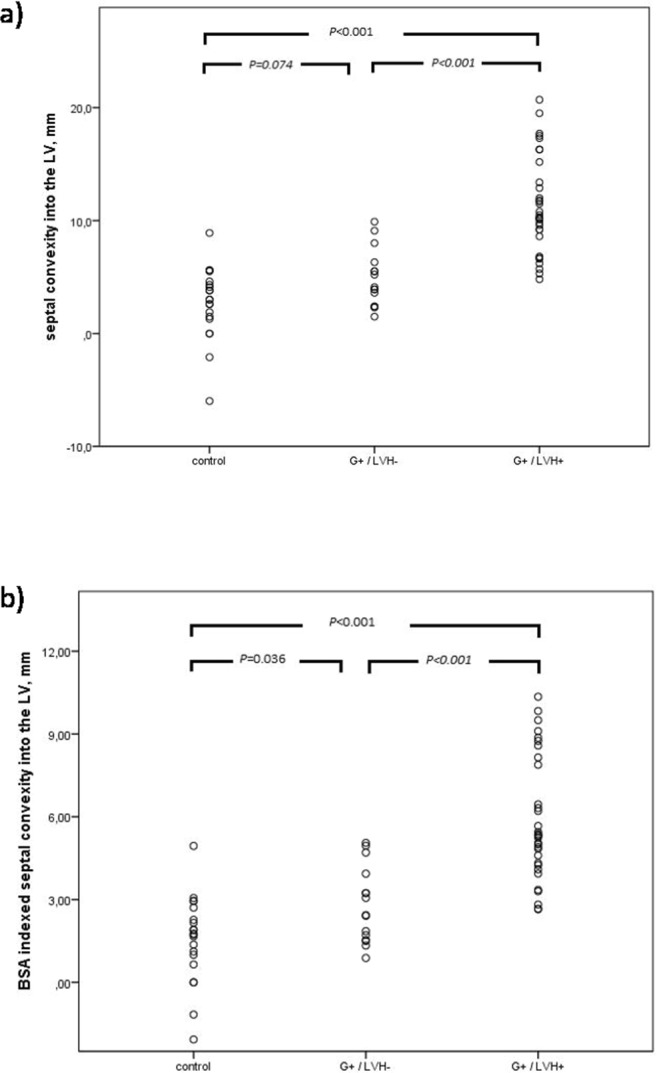
(**a**) Septal convexity into the LV in study groups. There is a significant difference between the G+/LVH+ group and other groups. (**b**) Septal convexity into the LV indexed for BSA. There is a significant difference between the G+/LVH+ group and other groups. Also the difference between G+/LVH− and control group is significant.

**Figure 3:**
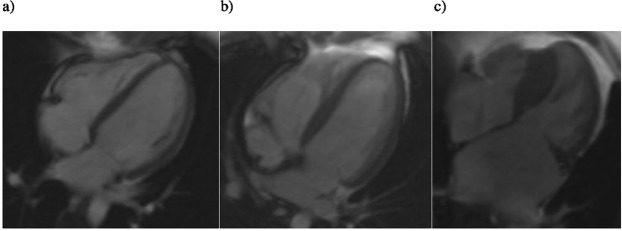
Ventricular end-diastolic CMR images in 4-chamber orientation (**a**) a control subject, (**b**) a subject with *MYBPC3*-Q1061X mutation but no LVH and (**c**) a subject with *MYBPC3*-Q1061X mutation and LVH.

Septal convexity measurement was feasible in all study subjects. Table 1 and Fig. 2a,b show the results of septal convexity measurements in the three study groups. There was a significant difference in septal convexity between G+/LVH+ group and controls (11.4 ± 4.3 vs 2.7 ± 3.2 mm, *P* < 0.001), and between G+/LVH+ and G+/LVH− groups, respectively (11.4 ± 4.3 vs 4.9 ± 2.5, *P* < 0.001) (Table 1 and Fig. 2a) (Fig. 3). The difference between the G+/LVH− group and the controls was not quite significant (4.9 ± 2.5 vs 2.7 ± 3.2 mm, *P* = 0.074; CI 95% 3.5–6.3 mm vs 1.2–4.2 mm). However, when indexed for BSA, the difference in SC values between all groups, including the G+/LVH− group and the control group, was statistically significant (2.8 ± 1.4 vs 1.5 ± 1.6 mm/m^2^, *P* = 0.036). Also the difference between G+/LVH+ group and controls (5.9 ± 2.2 vs 1.5 ± 1.6 mm/m^2^, *P* < 0.001) and G+/LVH+ group and G+/LVH− group (5.9 ± 2.2 vs 2.8 ± 1.4 mm/m^2^, *P* < 0.001) was significant (Fig. 2b).

In *MYBPC3* mutation carriers, increased septal convexity correlated significantly with BSA (r = 0.332, *P* = 0.006), age (r = 0.508, *P* < 0.001), LVMWT (r = 0.742 *P* < 0.001), LVM (r = 0.590, *P* < 0.001) and LGE (r = 0.683, *P* < 0.001). There was no correlation between septal convexity and LVEF (r = −0.083, *P* = 0.578) or LV volumes (LVEDV, r = 0.131, *P* = 0.379 and LVESV, r = −0.111, *P* = 0.459).

**Figure 4:**
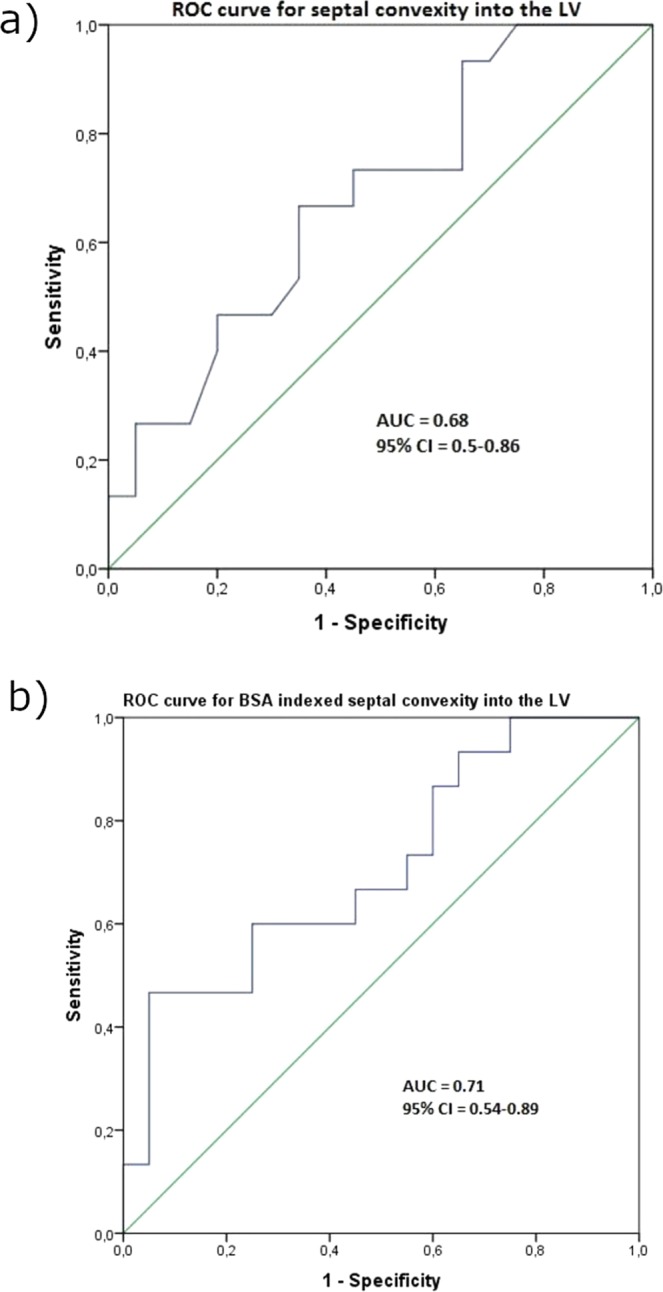
(**a**) ROC curve for septal convexity into the LV to differentiate between *MYBPC3*-Q1061X mutation carriers without LVH and controls. AUC is 0.68 (95% Confidence Interval (CI) 0.5–0.86). (**b**) ROC curve for BSA indexed septal convexity into the LV to differentiate between *MYBPC3*-Q1061X mutation carriers without LVH and controls. AUC is 0.71 (95% Confidence Interval (CI) 0.54–0.89).

Figure 4 shows receiver operating characteristic (ROC) curve of septal convexity as a predictor of mutation presence but no LVH. Area under the curve (AUC) was 0.68 and a cutoff value of 3.85 mm performed best with sensitivity 67% and specificity 65%. When indexed for BSA, AUC was 0.71 and a cutoff value 2.34 mm/m^2^ performed best with sensitivity 60% and specificity 75%.

### Reproducibility of septal convexity measurements

There was no significant difference between the first and second measurement of the septal convexity in intraobserver analysis measurements (10.3 ± 4.6 vs 10.5 ± 4.7 mm, *P* = 0.66) and the correlation between measurements was excellent (r = 0.961, *P* < 0.001). Interobserver measurements showed no significant difference between measurements (10.3 ± 4.6 vs 10.4 ± 4.2 mm, *P* = 0.65) and an excellent correlation between measurements (r = 0.973, *P* < 0.001).

## Discussion

Subjects with the *MYBPC3*–Q10961X mutation have increased left ventricular septal convexity irrespective of the presence of LVH. In mutation carriers, septal convexity correlates with BSA, age and CMR derived LV maximal thickness, mass and late gadolinium enhancement.

To our knowledge, there is only one previous study on CMR derived LV septal convexity in HCM-causing mutation carriers^11^. In aforementioned study, abnormal convexity was significantly increased in 36 phenotype negative carriers of HCM-causing mutations in multiple sarcomere genes, including *MYBPC3*, beta-myosin heavy chain (*MYH7*), troponin t (*TNNT2*), troponin I (*TNNI3*), myosin regulatory light chain (*MYL2*), myosin essential light chain (*MYL3*), tropomyosin (*TPM1*), and cardiac alpha-actin (*ACTC1*)^11^. In the present study, all mutation carriers had a single founder mutation of *MYBPC3*, and the septal convexity in LVH negative mutation carriers differed significantly from that of healthy controls only after indexing for BSA. In the present study, LV septal convexity with a cut point of >3.85 mm (AUC 68%, sensitivity 67% and specificity 65%) predicted *MYBPC3*-Q1061X mutation presence without LVH. Correspondingly, in the previous study, septal convexity cut point of >3.55 mm had best balance between sensitivity (77%) and specificity (89%) to predict mutation presence without LVH^11^. When indexed for BSA, LV septal convexity sensitivity was somewhat lower but specificity was higher (AUC 0.71 with cut point of >2.34 m/m^2^, sensitivity 60% and specificity 75%).

We found that septal convexity correlates significantly with BSA, age, and CMR derived LV maximal thickness, mass and late gadolinium enhancement in subjects with *MYBPC3*-Q1061X. To our knowledge, there are no previous correlation analyses on the topic.

Hypertrophic cardiomyopathy is characterized by asymmetric LV hypertrophy, most often located in the LV anterior septum or free wall. Bulging of septum, resulting in septal convexity, may precede increase in maximal wall thickness in the development of HCM, as suggested by the previous and present study. LV septal convexity correlated with BSA, age, LVMWT, LVM, and LGE in subjects with *MYBPC3*-Q1061X in our study. We propose that septal convexity reflects early-onset, body size and age -related septal remodeling in carriers of HCM-causing sarcomere mutations.

Measuring LV septal convexity in CMR is simple and highly reproducible, as shown by the present and the previous study. Septal convexity is measured in 4-chamber CMR images, which are usually included in CMR protocols used in clinical setting, and are therefore, readily available for measurement in subjects with HCM or LVH of unknown origin, and suspect carriers of HCM-causing mutations. Septal convexity, like most CMR parameters, correlates significantly with BSA. Consequently, indexing for BSA probably increases utility of septal convexity as shown by our study.

Our study population is relatively small. On the other hand, it is a cohort with a single HCM-causing mutation and, consequently, bias caused by multiple diverse mutations is avoided. *MYBPC3* mutations are the most common cause of HCM accounting for about 40% of all HCM cases globally. Furthermore, *MYBPC3*-Q1016X might be regarded to represent most *MYBPC3* mutations, as like two thirds of all *MYBPC3* mutations, it leads to the production of a truncated protein and, consequently, to haploinsufficiency, characterized by absence of mutant protein in myocytes^16–18^.

Non-indexed measurements showed a non-significant trend for septal convexity abnormality in mutation carriers without LVH compared to control subjects. However, the G+/LVH− subjects in the present study were younger and had a smaller BSA than control subjects, and both age and BSA correlated with septal convexity. BSA indexed values showed significant difference in septal convexity between G+/LVH− subjects and controls.

Even in the present era of targeted sequencing of large cardiomyopathy gene panels, the pathogenic or likely pathogenic mutation is found only in about 60% of HCM cases^9^. Age-related increase in penetrance of HCM implies that a proportion of clinically unaffected mutation carriers will develop overt cardiomyopathy later in life, and thus, precautionary long-term evaluation of normal healthy mutation carriers and relatives of patients without disease-causing mutation is recommended^19^. Hence, we need imaging methods to identify the relatives at risk of developing HCM. Imaging is especially important in families with mutations which may represent LVH in the middle-ages, as in the case of *MYBPC3* mutations^2,20^. One previous study has suggested that CMR derived septal convexity is a potential tool in finding phenotype negative HCM -causing mutation carriers^12^. In the present study, we showed that CMR derived septal convexity is significantly increased in carriers of the single mutation in *MYBPC3*. Septal convexity was related to BSA, and particularly if indexed for BSA, differed between LVH-negative mutation carriers and controls. Consequently, CMR derived septal convexity may have value in the evaluation of family members of patients with HCM to find relatives at risk of developing clinical HCM, particularly when genetic testing in the proband is not available or is negative. As septal convexity correlated with age and LVH and myocardial fibrosis in the whole mutation carrier group, it might be useful measure of severity of the disease also in the follow-up of patients with overt HCM. However, as septal derived convexity has been investigated only in two small studies, its utility requires further large-scale studies.

As a conclusion, subjects with the *MYBPC3*–Q10961X mutation have increased septal convexity irrespective of the presence of LVH. Septal convexity appears to reflect early onset, body size and age-related septal remodeling and could be useful in identifying phenotype-negative mutation carriers.

### Ethical approval and informed consent

Institutional Review Board approval was obtained for the study and study methods were carried out in accordance with the relevant guidelines and regulations. Written informed consent was obtained from all subjects in this study.
